# Locoregional Therapy Pressure-Enabled Drug Delivery for Liver Cancers

**DOI:** 10.3390/cancers18152367

**Published:** 2026-07-23

**Authors:** Thomas Eggleston, Fady Bassem Fayek, Jacqueline Kowalke, Mina S. Makary

**Affiliations:** 1 Corewell Health Lakeland Hospitals—St. Joseph’s Hospital, St. Joseph, MI 49085, USA; 2 Sidney Kimmel Medical College, Thomas Jefferson University, Philadelphia, PA 19107, USA; fadybassem.fayek@students.jefferson.edu; 3 The Kentucky College of Osteopathic Medicine, Pikeville, KY 41501, USA; jlkowalke@outlook.com; 4 Division of Vascular and Interventional Radiology, The Ohio State University Wexner Medical Center, Columbus, OH 43210, USA; mina.makary@osumc.edu

**Keywords:** trans-arterial embolization, trans-arterial chemoembolization, trans-arterial radioembolization, interstitial fluid pressure, pressure-enabled drug delivery

## Abstract

Hepatic malignancies account for a substantial portion of global cancer mortality, with hepatocellular carcinoma (HCC), intrahepatic cholangiocarcinoma (IHC), and metastatic liver disease representing the most common diagnoses. While surgical resection and liver transplantation remain curative options for eligible patients, most patients are diagnosed at stages unsuitable for surgery. This has shifted medical management towards locoregional therapies (LRTs) which are often catheter-directed. Of these interventions, the use of conventional end-hole catheters for therapeutic infusion has been a mainstay of treatment, but this method is constrained by retrograde particle escape and elevated tumoral interstitial fluid pressure. Together, these factors limit drug penetration into the tumor microenvironment. Pressure-enabled drug delivery (PEDD), achieved through balloon-occlusion or microvalve-based catheter platforms, has emerged as a strategy to overcome these limitations. This narrative review synthesizes current evidence regarding PEDD and contextualizes its role within the broader LRT landscape. Preclinical studies and early clinical data illustrate improved drug-delivery characteristics, acceptable safety profiles, and highlight the potential for adaptation to regional immunotherapy regimens. However, while PEDD represents a promising advance in catheter-based hepatic oncologic therapy, prospective randomized comparisons against conventional infusion remain limited, and significant investigation is needed to establish its definitive role in interventional oncology.

## Simple Summary

Hepatic malignancies remain one of the most common and deadly cancers worldwide, particularly when surgical resection is not an option. For these patients, locoregional therapies have been adapted to deliver treatment directly to liver tumors using catheters placed in the blood vessels feeding the tumor. While this targeted approach is less invasive, drugs and treatment particles often spread unevenly or leak backward into healthy tissue. A newer paradigm, called pressure-enabled drug delivery, uses specialized catheter designs that generate sustained pressure during infusion. This method allows for more effective penetration into tumor blood vessels while reducing backward leakage. This review summarizes current catheter-based liver cancer treatments, explains how pressure-enabled drug delivery works, and examines emerging evidence supporting its use.

## 1. Introduction

In the case of unresectable hepatic cancers, both primary and metastatic tumors are often treated with locoregional intra-arterial therapies. The goal of these therapies is to deliver the desired treatment to the tumor and induce necrosis of the neoplastic cells in that region, all while sparing the surrounding, healthy parenchymal tissue [1]. The resistance of the tumor environment to said therapy can be described and quantified by an elevated interstitial fluid pressure (IFP). IFP is a multifactorial phenomenon that opposes the hydrostatic pressure in the vasculature and prevents extravasation of chemotherapeutic agents. This hostile environment redirects blood flow to tumor margins and results in non-homogeneous distribution of chemotherapy [2].

Pressure-enabled drug delivery (PEDD) is an emerging technology that leverages dynamic pressure modulation to increase the perfusion of therapeutic agents within the tumor, prevent reflux, and minimize damage to surrounding parenchyma [3]. Specifically, PEDD devices utilize one-way valves that adapt their orifice size in response to pressure gradients, allowing variance of flow rates at different stages of the cardiac cycle. This paper provides a comprehensive overview of investigations regarding the adaptation of PEDD for the treatment of hepatic tumors in the context of modern locoregional therapies (LRTs). Various evidence-based clinical and therapeutic advantages of this modality, when combined with conventional intra-arterial treatment modalities, are covered. Additionally, the synergistic use of PEDD with immunotherapy is discussed. A review on current clinical trials evaluating the deployment of PEDD for hepatopancreaticobiliary malignancies is further explored.

## 2. Current Catheter-Directed Locoregional Therapies

### 2.1. Trans-Arterial Embolization (TAE)

Trans-arterial embolization (TAE) is a bland embolization technique that induces selective hypoxia and tumor necrosis through the delivery of embolic agents [4,5]. Under fluoroscopic guidance, these agents are injected into tumor-feeding vasculature until arterial stasis is achieved, capitalizing on the vascular demands of the tumor which are grossly dependent on the hepatic artery instead of the portal vein like much of the liver parenchyma [6]. Agent selection remains debatable as it is a multifactorial decision dependent on tumor size, patient-specific flow patterns and anatomy, underlying liver function, and the required depth of vascular penetration [4,7,8,9].

Considerations for TAE primarily include intermediate-stage HCC, liver-dominant neuroendocrine metastases, and other arterial-dependent or hypervascular hepatic tumors [5,10,11]. In combination with systemic therapy, TAE can also be utilized for advanced or unresectable disease [8,12]. In either case, the goals of treatment are local control, downstaging, or as a bridge to transplantation in candidate patients [11]. TAE is, however, relatively contraindicated in decompensated cirrhosis (Child-Pugh C and Barcelona Clinic Liver Cancer (BCLC) stage D) and clinically symptomatic end-stage cancer [9,11]. Uncorrectable coagulopathy and portal vein thrombosis (PVT), wherein the liver becomes predominantly dependent on arterial blood supply, remain absolute disqualifiers [13,14,15]. Further, a wide variety of hepatic and extrahepatic complications have been noted post-procedurally in the medical literature, including liver abscess, femoral artery pseudoaneurysms, cholecystitis, pulmonary embolism, and variceal bleeding [13,16].

### 2.2. Trans-Arterial Chemoembolization (TACE)

Trans-arterial chemoembolization (TACE) is a descendant of TAE that utilizes arterial occlusion to cut off blood supply while simultaneously supplying chemotherapeutics to the local tissue. This provides a synergistic effect of ischemic necrosis and tumor cytotoxicity [17,18]. TACE can further be subdivided into three groupings: conventional TACE (c-TACE), drug-eluting bead TACE (DEB-TACE), and degradable starch microsphere TACE (DSM-TACE). Each offers a unique therapeutic modality in the context of LRTs. c-TACE involves mixing the chemotherapy agent(s) with radiopaque lipiodol for real-time monitoring of treatment delivery. Alternatively, DEB-TACE employs non-absorbable microspheres that continuously release chemotherapeutic agents while also embolizing the tumor’s arteriolar supply [14]. Compared to c-TACE, DEB-TACE has been found to have lower systemic concentrations of infused chemotherapeutics while synchronously increasing chemotherapy concentrations at tumor sites [19,20]. A newer regimen, DSM-TACE utilizes biodegradable microspheres that degrade within 30–60 min of introduction. This eliminates the permanent ischemia introduced by other TACE techniques and, in turn, reduces the release of HIF-1a and VEGF that promote tumor neoangiogenesis and progression [21,22,23,24,25]. Interestingly, several clinical trials have investigated the use of TACE in combination with immunotherapy and targeted therapy. These studies have further demonstrated improvements in tumor responses and progression-free survival for treatment-resistant HCC [26,27].

Unlike TAE, TACE is considered the standard of care for intermediate (BCLC stage B) HCC without vascular invasion, extrahepatic spread, or significantly decreased liver function (beyond Child-Pugh A–B) [18]. TACE can also be considered in select patients who are not candidates for, or have progressed on, systemic therapy [8,15,28,29,30]. The goal of treatment, however, remains a bridge therapy to liver transplantation, downstaging, or local control [11,31,32,33]. TAE and TACE also share similar contraindications, such as decompensated cirrhosis (Child-Pugh C), clinically symptomatic portal hypertension, tumors > 10 cm, widespread bilobar tumor burden, and uncontrolled biliary obstruction [9]. As in all cases, overall survival and adverse effects seen in HCC patients being treated with TACE continue to be related to tumor size, number, and the agent selected [34,35].

### 2.3. Trans-Arterial Radioembolization (TARE)

Trans-arterial radioembolization (TARE) is a local radiation therapy that delivers glass or resin-based yttrium-90 (Y-90) microspheres, a β-emitting radioisotope, to hepatic tumors via the hepatic arteries. This allows for the delivery of high-dose radiation to various hepatic tumors without significantly impacting the surrounding hepatic parenchyma [36,37]. Prior to therapy, hepatic mapping angiography is conducted using technetium-99 m (^99m^Tc) macro-aggregated albumin (MAA) particles which simulate the deposition of radioactive microspheres and help to identify potential off-site sphere deposition [36,38]. This protocol allows for something quite unique to the TARE treatment modality, personalized dosimetry, or the tailoring of dosing to a patient’s specific tumor burden and anatomy. Specifically, personalized dosimetry allows for larger radiation doses to be delivered to the mapped tumor, which in concept would produce a more robust tumor response. Compared with standardized dosimetry, personalized dosimetry has been shown to improve objective response rates while maintaining a favorable adverse-event profile [39].

In general, the indications and contraindications of TARE are congruent with those of TACE and TAE for primary and metastatic hepatic disease [14,37]. However, TARE has further demonstrated effectiveness in early-stage and unresectable (BCLC A) HCC, unresectable intrahepatic cholangiocarcinoma, and for patients with PVT in whom further embolization would risk hepatic decompensation [36,40,41,42,43]. TARE has also been heavily utilized in the context of early-stage, solitary tumors < 5 cm which are anatomically difficult to ablate and as an alternative to portal vein embolization (PVE) [44,45]. This utility is, however, somewhat limited given the need for prophylactic embolization, and the possibility for spread of the therapy to nontarget vasculature, often including pulmonary vasculature, gastroduodenal, and right gastric arteries [46,47]. Y-90 microspheres, specifically those that are resin-based, can persist in the gastroduodenal tract for years and cause refractory ulceration [48].

### 2.4. The Barcelona Clinic Liver Cancer (BCLC) Framework

In general, therapies for hepatic malignancies follow established clinical staging frameworks. The most prominent of these classifications is the BCLC, which integrates tumor burden, liver function, and performance status in order to direct treatment selection [12,44]. Within the framework, surgical resection and transplantation deliver the strongest curative-intent outcomes for patients with limited tumor burden and adequate hepatic reserve. However, these interventions become less feasible as tumor multifocality, vascular invasion, or hepatic dysfunction develop. Ablation extends this curative intent to small lesions in non-surgical candidates, but it remains limited by many of the same variables. In these desperate cases, systemic therapy becomes the principal modality. Each modality has a stage-appropriate role rather than competing for the same patient population, and patients often progress across multiple treatment categories during the course of their disease (Table 1) [12,44].

The standard of care niche for catheter-directed therapies, as described above, are the BCLC-endorsed intermediate-stage malignancies, meaning that PEDD is used as a delivery refinement for LRTs which already occupy validated positions in the BCLC algorithm, not as a substitute for surgical, ablative, or systemic options. It is intended to enhance the efficacy of treatments at the intermediate stage and bridging indications where LRTs already serve as the standard of care while preserving the multidisciplinary pathway that integrates all available modalities.

## 3. The Evolution of Catheter-Based Delivery Platforms

**Figure 1:**
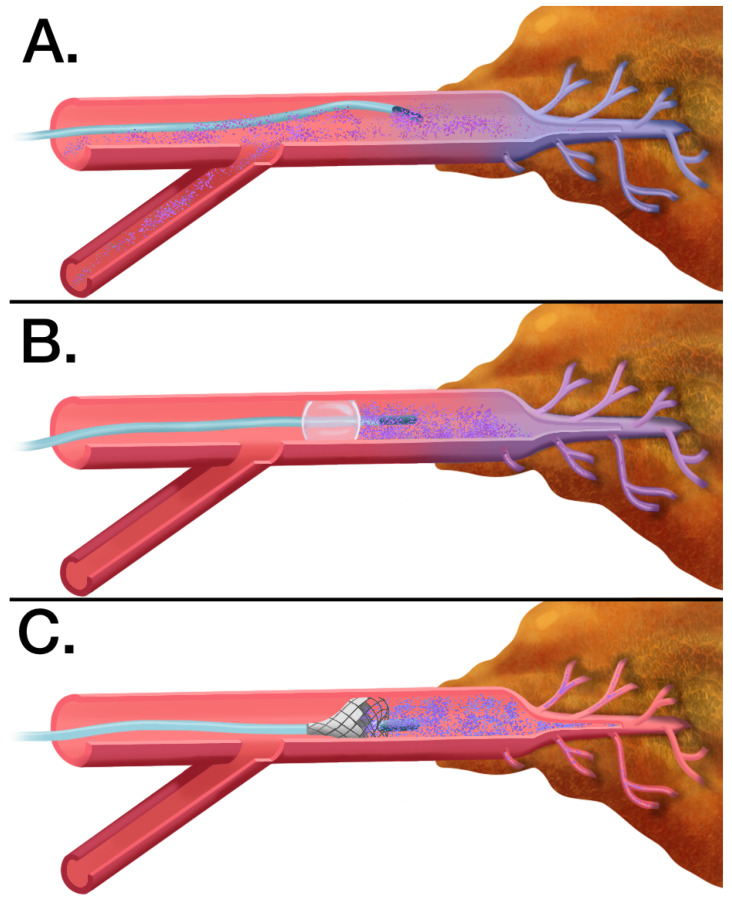
Illustrative comparison of the three catheter-based delivery platforms during intra-arterial infusion of a hepatic tumor: (**A**) End-hole microcatheter: antegrade infusion of the therapeutic agent (purple-blue particulates) occurs without resistance to retrograde particle escape or propulsion against elevated IFP (blue vessel coloration); (**B**) Balloon-based catheter: temporary inflation of a proximal microballoon arrests antegrade flow and redistributes embolic material toward tumor-feeding vessels; (**C**) Microvalve-based catheter: a collapsible and expandable tip preventing reflux and generating a sustained local pressure gradient at the site of IFP.

While revolutionary, the therapies described in the preceding section depend on the ability to deliver embolic and chemotherapeutic agents specifically to target tissues at effective concentrations. This step remains a persistent technical and physiological challenge, as these agents frequently fail to permeate the interstitial space of solid tumors. This is, in part, because the IFP within the tumor is elevated well above that of the surrounding parenchyma [2,49,50]. Multiple physiologic characteristics common to solid tumors work to maintain this pressure gradient, including aberrant angiogenesis, underdeveloped intratumoral lymphatics, and compressive solid stress [49,50]. The resulting hydrostatic barrier opposes uniform drug penetration and redirects blood flow toward the tumor periphery [2,50]. In vitro studies have further shown that elevated IFP in and of itself promotes hepatocellular carcinoma proliferation, metastasis, and resistance to apoptosis, creating a pro-oncogenic loop that compounds the delivery problem [51]. These observations have driven the development of catheter-based platforms that aim not only to navigate complex anatomy but to also modulate local hemodynamics in ways that improve intratumoral drug deposition (Figure 1).

### 3.1. End-Hole Catheters

The standard platform for intra-arterial liver-directed therapy is the end-hole microcatheter. Devices such as Progreat (Terumo, Somerset, NJ, USA) and Renegade (Boston Scientific, Marlborough, MA, USA) were designed for navigation of the tortuous hepatic vasculature, but their open-ended configuration imposes no resistance to retrograde particle escape [52]. Reflux of embolic material into nontarget territories, such as the gastroduodenal and right gastric arteries, has historically required prophylactic coil embolization to prevent gastrointestinal mucosal injury [52]. This means that additional intervention and skillful titration of the infusion rate under fluoroscopy are required to mitigate reflux. However, this is a subjective endpoint that does not account for the downstream hemodynamic conditions within the tumor. Drug mixing is also often incomplete because agents are subject to laminar flow within the vessel rather than being consistently dispersed across the lumen, which yields an uneven delivery across downstream branch vessels [53]. Two distinct catheter designs have emerged to address these limitations, balloon-based occlusion platforms and, more recently, microvalve-based platforms.

### 3.2. Balloon-Based Catheters

These devices use a temporarily inflated microballoon to arrest antegrade flow and establish a pressure-isolated vascular compartment at the infusion site. Devices such as the Attendant (Terumo) or Logos (Piolax) microballoon catheters are positioned and inflated proximally to the tumor. Post-inflation and arrest of antegrade flow, a measurable drop in distal arterial pressure promotes embolic agent movement toward tumor-feeding vessels through a favorable pressure gradient [54,55]. This system, B-TACE, has demonstrated significantly higher rates of complete tumor necrosis when compared to conventional TACE, with, at most, a transient increase in hepatic dysfunction [56,57]. The first direct comparative study of selective B-TACE versus selective conventional TACE identified B-TACE as an independent predictor of both improved primary nodule control and improved overall survival on multivariate analysis [58]. Mechanistic in vivo work by Lucatelli et al. further confirmed that balloon occlusion increases intratumoral deposition of both drug-eluting microspheres and yttrium-90 [59]. This is a finding corroborated in a European propensity-score-matched multicenter analysis by Golfieri and colleagues, as well as a single-center case–control analysis by Lucatelli et al., wherein both reported higher complete response rates and lower retreatment rates with B-TACE than with conventional or DEM-TACE [60,61]. B-TACE has also been applied successfully to large HCC nodules, with repeated alternate infusion of cisplatin and gelatin slurry, achieving objective responses in all 19 nodules greater than 7 cm and a 58% complete response rate [62].

A related balloon-based device, the Sniper Balloon Occlusion microcatheter (Embolx, Sunnyvale, CA, USA), is positioned distally and inflated proximal to the infusion site, generating a negative-pressure zone beyond the balloon that can reverse flow in collateral vessels and redirect the desired agents toward tumor-feeding branches. Applications of this technique have also been noted to capitalize on the reversal of flow in the diaphragmatic arteries during TACE to enhance hepatic tumor delivery [63]. Building on these comparative outcomes, recent single-center evidence further supports B-TACE as a potentially curative-intent option in selected patients. In 82 treatment-naive HCC patients undergoing lipiodol-based subsegmental B-TACE, Gwon and colleagues observed an initial complete response rate of 98.8% and a curative response in 78% of patients over a median 39.1-month follow-up [64].

### 3.3. Microvalve-Based Catheters

Rather than using an inflatable balloon, these devices incorporate a collapsible, funnel-shaped expandable tip distal to the infusion port. During systole, antegrade blood flow partially collapses the tip to permit particle transit into the tumor. During diastole, and in response to any retrograde flow, the tip expands to occlude the vessel lumen and prevent reflux. Importantly, because the microvalve modulates flow rather than arresting it, antegrade perfusion is preserved throughout the infusion. Rose et al. demonstrated in 18 patients undergoing hepatic embolization that deployment of the expandable tip produced a mean downstream arterial pressure decrease of 22 mmHg (*p* < 0.00001). This effectively partitions the vascular compartment of interest from the greater systemic circulation and produces favorable downstream effects similarly seen in the balloon-based devices as discussed above [65]. This marks a distinct mechanistic difference from balloon-based devices as balloon-occluded delivery systems arrest antegrade flow and rely on the resulting hemodynamic drop to redirect embolic material into tumor-feeding branches. Microvalve-based devices, however, preserve systolic antegrade flow and use the retained forward pressure to drive therapeutic agents into the hostile tumor microenvironment. Additionally, bench-top modeling published by van den Hoven and colleagues showed that the expandable tip promotes a more centralized catheter position while inducing turbulent rather than laminar flow. This produces significantly more homogeneous downstream particle distribution than a standard microcatheter [53]. Whether these complementary mechanisms produce additive or comparable clinical benefit remains untested.

## 4. Mechanism of Pressure-Enabled Drug Delivery

PEDD describes the use of microvalve-based catheters to generate and maintain a more favorable local pressure gradient during infusion while preventing reflux. Specifically, deployment of the microvalve initially reduces downstream arterial pressure by restricting flow through the device [65]. Then, during active infusion both antegrade blood flow during systole and progressive distal embolization raise pressure within the partitioned compartment [66]. It is this partitioned and pressurized compartment that acts against the tumor IFP gradient. The TriNav Infusion System (TriSalus Life Sciences, Westminster, CO, USA) exemplifies this approach, wherein the infusate is delivered against the partially occluded vessel so that forward flow is retained while retrograde escape is prevented [67].

### 4.1. Overcoming Intratumoral Interstitial Fluid Pressure

As discussed previously, solid tumors generate elevated IFP through several interrelated mechanisms. Aberrant angiogenesis produces both structurally defective vasculature and poor-to-absent lymphatic drainage that allows for interstitial fluid accumulation [2,49,50,68]. Stromal fibrosis and uncontrolled cellular proliferation also exert compressive solid stress that physically collapses intratumoral vessels; producing a radially decreasing pressure gradient from the tumor core to its periphery [49,50,51]. Shankara Narayanan et al. characterized this barrier directly in an orthotopic murine pancreatic adenocarcinoma model. This study showcased a baseline tumor IFP averaging at 45 mmHg. Pressure-enabled retrograde venous infusion of gemcitabine then raised local IFP by a further 29 mmHg and yielded roughly 7-fold higher intratumoral drug concentrations than systemic delivery (127 vs. 19 ng/mg, *p* < 0.01) [69]. These data support the concept of restrictive IFP and would suggest that externally applied hydraulic pressure can reopen collapsed tumor microvasculature to restore drug penetration into regions that are pharmacologically inaccessible under strained hemodynamic conditions.

### 4.2. Integrating Pressure, Flow, and Vascular Dynamics

Compared to traditional end-hole catheters, the gains observed with PEDD reflect three advancements in combating the hemodynamic effects previously discussed. First, the dynamic microvalve centers the catheter tip within the vessel lumen. This reduces wall-contact streaming and improves infusion consistency [67]. Second, flow over the valve generates turbulence at the distal tip, promoting mixing of particles with blood and a more even distribution across downstream branch vessels. This was most notably seen in a comparison study by van den Hoven and colleagues, in which bench-top models illustrated a downstream heterogeneity deviation of 15.5% with the ARC modality compared to a 40.9% deviation with an end-hole catheter (*p* = 0.047) [53]. Third, reflux prevention allows pressure to build locally within the target arterial network. Rose and colleagues showed that this pressure progressively increases during TACE as downstream embolization accrues. This in turn produces a measurable systemic-to-hepatic arterial pressure difference (SHAPD) that varies with embolic type and degree of obstruction [66]. This specific mechanism is further enhanced by the aberrant angiogenesis of tumor vessels, as these vessels typically lack the neural and smooth muscle compartments present in normal vasculature. This means that, instead of vasoconstricting in response to sudden hypotension, these vessels remain grossly patent, further redistributing blood and chemotherapeutics towards the tumor. Together these effects generate the sustained local pressure gradient that drives particles against the IFP barrier and into previously inaccessible tumor vasculature.

### 4.3. Preclinical PEDD Microsphere Delivery

While these mechanistic advancements substantiate the potential benefit of PEDD, much of the demonstrable data remain preclinical, and the reported gains serve as surrogate markers of delivery rather than guideline-directed oncologic endpoints. Namely, two Oncopig studies published by Jaroch et al. tested the PEDD hypothesis in vivo, using particles representative of the two principal hepatic embolotherapy classes. In these cases, fluorescently labeled glass microspheres (19.3 to 20.7 µm) acted as surrogates for Y-90 spheres [67], and larger trisacryl gelatin embolic microspheres (100 to 300 µm) were substituted for those used in drug-eluting bead TACE [70]. Near-infrared imaging with a custom deep-learning algorithm quantified signal intensity in concentric 1 mm zones extending both into and away from the tumor border.

In the case of glass microspheres, lobar PEDD (*n* = 10) increased total intratumoral penetration by 117% over conventional end-hole delivery (*n* = 7, *p* = 0.004), with higher signal across every zone from 20 mm inside the tumor core to 10 mm beyond the border (*p* < 0.05 for all zones). Selective PEDD (*n* = 9) produced a 39% increase over end-hole controls (*n* = 8, *p* = 0.032). Importantly, delivery to normal liver beyond 30 mm from the tumor margin did not differ between groups. This would suggest that improved drug delivery was not achieved at the expense of off-target parenchymal exposure. Lobar PEDD also produced therapeutic delivery equivalent to conventional selective delivery (*p* = 0.497), meaning that this modality of treatment is comparable to conventional methods. Clinically, multifocal or infiltrative disease treated via a single proximal catheter position may achieve what currently requires time-consuming catheterization of individual feeding vessels without loss of efficacy [67].

For larger embolic microspheres, selective PEDD (*n* = 8) increased total intratumoral penetration by 227% over conventional delivery (*n* = 8, *p* = 0.029) and peritumoral delivery within 5 mm of the tumor border by 209% (*p* = 0.045). The tumor-to-normal tissue (T/N) ratio improved from 2.7 to 4.2 and the peritumor-to-normal (PT/N) ratio from 3.1 to 4.6. A trend toward reduced normal tissue exposure in the treated angiosome (*p* = 0.090) accompanied these gains [70]. Again, this follows the trend of improving therapeutic treatment without sacrificing the established effectiveness or precision of traditional modalities.

## 5. Clinical Evidence and Therapeutic Advantages of PEDD

Clinical translation of PEDD requires demonstration of safety, feasibility, and efficacy in patients. The published evidence comprises retrospective analyses, single-center series, real-world cohorts drawn from industry-sponsored datasets, and early-phase immunotherapy trials (Table 2). However, while the available data consistently show improved drug-delivery characteristics with an acceptable safety profile, prospective randomized comparisons against conventional delivery remain limited.

### 5.1. Improved Intratumoral Drug Delivery

The earliest histopathological evidence of improved delivery came from Titano and colleagues, who retrospectively compared microvalve infusion (MVI) to end-hole delivery during drug-eluting microsphere TACE for solitary HCC [71]. In explant pathology from a total of 23 patients (*n* = 5) who underwent liver transplantation, the MVI group demonstrated 88.7% on-target microsphere deposition versus 55.3% for the end-hole technique. Corresponding tumor necrosis was also noted to be 89% versus 56% for each group, respectively. These findings remain the only explant-level validation of improved delivery with a microvalve catheter and provide a histopathologic grounding for the preclinical fluorescence findings of both the Jaroch studies and the vasoreactivity postulation of Pasciak et al. [67,70,72]. Kim and colleagues separately reported on 22 patients with unresectable HCC treated with DEM-TACE via the Surefire Infusion System (SIS), observing a 32% per-patient and 54% per-lesion complete response rate after a single session [73]. These rates compare rather favorably to historical end-hole DEM-TACE series of 15 to 25% for comparable tumor sizes.

### 5.2. Decreased Reflux and Nontarget Embolization

Prevention of nontarget delivery was the original design intent of the SIS/TriNav platform, and, as such, the clinical evidence in this domain is the most robust. To this point, Fischman et al. demonstrated in a prospective RCT that using an antireflux microcatheter during planning angiography eliminated the need for prophylactic coil embolization while achieving comparable prevention of nontarget distribution and significantly reduced fluoroscopy time, procedure time, and radiation dose [74]. Van den Hoven and colleagues confirmed exclusive intrahepatic microsphere deposition in patients treated without prior coil embolization on post-treatment PET-CT [75]. These observations are further supported preclinically by Arepally et al., wherein an ex vivo analysis of renal artery embolization with tantalum microspheres and microCT quantification in swine demonstrated 99.9% ± 1.0% embolization efficiency with the ARM versus 72% ± 13% with a conventional catheter. This represents a significant decrease in nontarget embolization (*p* < 0.05) [76]. A cautionary counterpoint comes from a case report of a biloma following bland embolization with the TriNav device, in which the authors hypothesized that enhanced distal embolic penetration may increase ischemic exposure of the peribiliary plexus [77]. This highlights the continued need for careful titration of embolic endpoints, specifically in the context of bland embolization and small-caliber particles.

### 5.3. Sparing of Normal Hepatic Parenchyma

Preservation of functional liver reserve is critical in patients with hepatic malignancy, many of whom have an innate complexity of underlying cirrhosis, prior treatment exposure, or limited hepatic reserve. The differential vasoreactivity observed by Pasciak and colleagues, wherein there was simultaneously reduced nontarget embolization and increased tumor deposition in all nine patients (*p* < 0.05), provides a likely biophysical basis for improved selectivity [72,78]. Consistent with this, neither of the Jaroch studies showed increased microsphere deposition in normal liver beyond 30 mm from the tumor border, and the embolic-microsphere study demonstrated a trend toward reduced cumulative normal-tissue exposure in the treated angiosome (*p* = 0.090). As previously mentioned, a 56% improvement in the T/N ratio from 2.7 to 4.2 was observed [67,70]. Because treatment-related decompensation remains a concern in cases of serial TACE or TARE treatments, a delivery platform that concentrates the therapeutic effect within a tumor while minimizing collateral damage could extend the treatment window for patients requiring multiple sessions [44]. However, this is a hypothesis not yet tested prospectively.

### 5.4. Safety and Economic Outcomes

The largest clinical dataset evaluating PEDD in routine practice was published by Gupta et al. as a retrospective industry-affiliated analysis [79]. Using the Clarivate Real World Data repository, the authors identified 603 PEDD patients and 16,210 conventional-technique controls treated between January 2020 and March 2024. As in the earlier claims-based analysis by Cook and colleagues, patients selected for PEDD had higher Charlson comorbidity indices and greater disease burden than the conventional cohort [80]. In matched analyses, PEDD was associated with reduced post-procedure fatigue (20.9% vs. 26.4%, *p* < 0.05) and a 61% reduction in 30-day inpatient visits among TACE recipients (8.0% vs. 20.5%, *p* < 0.05). Effects were more pronounced in “high-adopter” facilities, where PEDD was associated with lower overall lymphopenia (0.6% vs. 5.2%; *p* < 0.05). Among patients with secondary liver metastases at these facilities, PEDD was associated with substantially lower rates of fatigue (19.2% vs. 39.7%) and lymphopenia (0.0% vs. 8.2%) (*p* < 0.05 for both). This is a pattern grossly consistent with a learning-curve effect.

Gupta et al. estimated a per-patient charge avoidance of $7734 with PEDD ($4599 from fewer complications, $3135 from reduced inpatient stays), while Morshedi et al. previously documented approximately $7000 in per-case savings from eliminating coil embolization during TARE planning [79,81]. Although the two estimates are not directly additive, they could indicate that PEDD’s higher per-procedure device cost may be offset by reductions in downstream healthcare resource use.

### 5.5. PEDD as a Platform for Regional Immunotherapy

Immunotherapy has emerged as a key field of study in the development of therapeutics designed to combat multiple cancerous pathologies. The liver microenvironment is somewhat unique in that it maintains a quality of immunosuppression mediated by myeloid-derived suppressor cells (MDSCs). These cells expand in response to tumor colonization and suppress cytotoxic T-cell function through the GM-CSF/STAT3/IDO/PD-L1 axis [82,83,84]. This quality is organ-specific and pharmacologically reversible, possibly opening the door to regional reprogramming of the hepatic immune compartment [84,85].

Capitalizing on this unique physiology, the Katz group has led clinical development of regional cellular immunotherapy through the Hepatic Immunotherapy for Metastases (HITM) program. Phase I HITM established the safety and feasibility of anti-CEA CAR-T delivery via hepatic arterial infusion for CEA-positive adenocarcinoma liver metastases [86]. The Phase Ib HITM-SIR trial combined CAR-T with Y-90 SIRT in six heavily pre-treated patients, with no grade 4 or 5 toxicities, no severe cytokine-release syndrome, a mean 48% decrease in serum CEA, and one metabolic complete response on PET [87]. The HITM-SURE case report then provided the first direct linkage between PEDD-enhanced delivery and a clinical outcome. Here, a patient receiving anti-CEA CAR-T via the TriNav device in PEDD mode achieved an estimated 5.2-fold increase in CAR-T delivery and a 13-month complete response without adverse events above grade 3 [88]. This work would suggest that the implementation of PEDD in future immune-based therapy could be highly effective; however, as a single-patient observation, this finding serves more as a clinical anchor rather than definitive evidence.

Specifically, preclinical work has extended this paradigm to immunomodulatory agents. PEDD-delivered TLR9 agonist combined with anti-PD-1 blockade reduced tumor burden in a murine pancreatic adenocarcinoma model, with pressurized regional venous infusion yielding an approximately 3-fold higher intratumoral signal than systemic delivery [84,89]. PEDD hepatic arterial delivery of nelitolimod (SD-101) was feasible in both Oncopig and murine models, reducing tumor progression, depleting intrahepatic MDSCs, and promoting cytotoxic CD8^+^ T-cell responses [3]. Additionally, co-administered regional TLR9-agonist delivery and systemic anti-PD-1 depleted intrahepatic MDSCs and activated antitumor immunity, with the combination outperforming either agent alone [90]. IL-10 blockade, another target in the hepatic immunosuppressive program, produced a 1.8-fold increase in carcinoma cell death in human colorectal liver metastasis slice cultures and enhanced anti-CEA CAR-T cytotoxicity [91]. Collectively these data support using PEDD to concentrate immunomodulatory agents within the hepatic tumor microenvironment and shift it toward a state permissive of antitumor immunity [3,82,84,89,90]. However, phase II or III data confirming oncologic benefit are not yet available.

## 6. Future Directions

The clinical trial landscape for PEDD has expanded rapidly (Table 3). The TriSalus-sponsored PERIO program anchors the industry-led effort, with three trials evaluating PEDD-delivered nelitolimod across uveal melanoma liver metastases (PERIO-01), HCC and intrahepatic cholangiocarcinoma (PERIO-02), and locally advanced pancreatic adenocarcinoma (PERIO-03). PERIO-01 and PERIO-02 were terminated during enrollment, reflecting the broader challenges of early-phase oncology trials in rare indications. However, the active PERIO-03 trial may provide a prospective clinical assessment of PEDD-delivered immunotherapy in a solid tumor.

Investigator-initiated trials are addressing complementary questions. PEDIR (Massachusetts General Hospital, Boston, MA, USA) and the TriNav Fidelity Study (University of Texas, MD Anderson, Houston, TX, USA) are primed to evaluate whether PEDD improves concordance between pre-treatment ^99m^Tc-MAA mapping and post-treatment Y-90 distribution. PEDD-cTACE (Abramson Cancer Center, Penn Medicine) randomizes HCC and neuroendocrine liver metastasis patients to end-hole versus TriNav delivery during lipiodol TACE, quantifying intratumoral lipiodol volume and density on post-procedure CT. This represents the first randomized imaging-based comparison of PEDD in the TACE setting and a clinical analog to the Jaroch preclinical microsphere studies and the Titano explant analysis [67,70,71]. Additional trials, including PREDICTT at MD Anderson and a UC San Diego trial combining cryoablation with PEDD-delivered SD-101 and dual checkpoint inhibition (STRIDE regimen), represent a growing interest in multimodal protocols that use PEDD as a delivery platform.

However, several questions remain unresolved. Of the greatest clinical interest are perhaps the ideal infusion rate, pressure thresholds, and catheter positioning relative to tumor-feeding vessels. Preclinical data also suggest that benefits may vary with tumor vascularity. Specifically, hypervascular lesions, such as HCC, may derive greater benefit than hypovascular metastases. Consistent with this, a within-patient randomized trial of an antireflux catheter during ^166^Ho-radioembolization in colorectal liver metastases (typically hypovascular) found no improvement in tumor-to-nontumor activity ratios [92]. However, the mechanistic prerequisite for pressure-augmented delivery is a measurable downstream pressure reduction upon device deployment, and this physiologic gradient may be blunted by concurrent vasoactive premedication [93]. Whether the null finding reflects a true absence of benefit in hypovascular disease or an unrecognized attenuation of the pressure differential remains unresolved. Institutional experience also appears to influence outcomes [80]. The additive or synergistic value of PEDD relative to B-TACE, which shares some hemodynamic principles, has not been directly compared. Finally, most published PEDD efficacy data are derived from industry-sponsored preclinical studies or retrospective clinical analyses. Independent, prospective, randomized trials with oncologic endpoints are essential to establish the clinical value of PEDD definitively.

The convergence of PEDD with regional immunotherapy is seemingly of particular clinical interest. Chai and colleagues showed that regional anti-PD-1 delivery produces higher intratumoral concentrations and superior tumor control with reduced systemic exposure in murine models, and the Katz laboratory has systematically characterized the MDSC-driven program that blunts conventional systemic immunotherapy in liver metastases [3,82,84,85,89,94]. PEDD offers a technical solution to the delivery challenges that have historically limited regional approaches [3,89]. Integration into treatment algorithms will also require attention to cost. The TriNav system is more expensive per procedure than standard microcatheters, but data from Morshedi et al. and Gupta et al. suggest that elimination of coil embolization and reductions in post-procedure complications can offset much of this difference [79,81]. Whether improved delivery ultimately translates into fewer treatment sessions, reduced hospitalization, or longer progression-free intervals remains to be determined.

## 7. Conclusions

Elevated intratumoral IFP remains one of the most significant and underappreciated barriers to effective locoregional therapy in hepatopancreaticobiliary malignancies. Conventional catheter-based delivery systems were not designed to address this barrier directly. PEDD, implemented through the TriNav Infusion System, represents a potentially beneficial mechanistic shift. Rather than simply preventing reflux, the system actively generates a local pressure gradient that opposes intratumoral IFP, reopens collapsed microvasculature, and drives therapeutic agents into tumor tissue.

Preclinical data demonstrate consistent, statistically significant improvements in microsphere penetration with PEDD across both glass and embolic-sized particles, with lobar PEDD achieving delivery equivalent to conventional selective infusion. Clinical data, while predominantly retrospective, indicate that PEDD is safe in patients with complex profiles and improve pathologic tumor response during DEM-TACE. Real-world evidence confirms comparable or improved safety alongside reductions in post-procedure complications and healthcare utilization [79,80]. The application of PEDD to regional immunotherapy, including CAR-T cells and TLR9 agonists, capitalizes on the distinct immunobiology of the hepatic microenvironment [3,88,89,90,94]. These gains, however, remain surrogate measures of delivery, and the supporting clinical literature consists predominantly of retrospective analyses, single-center series, and industry-affiliated real-world studies. Prospective randomized trials comparing PEDD to both conventional and balloon-based delivery with oncologic endpoints are needed to move this technology from a promising innovation to an established component of interventional oncology.

## Figures and Tables

**Table 1:** Barcelona Clinic Liver Cancer (BCLC) Classifications and Reported LRT Outcomes [12,18,41,44].

**Stage**	**Standard**	**Outcome**
BCLC A (Curative-intent)	Resection, liver transplantation	5-year OS *: 60–80% (resection), 70–80% (transplant, within Milan criteria)
BCLC A (Non-surgical, <5 cm)	Ablation (RFA *, MWA *, cryoablation), SBRT * alternative	1- and 3-year OS: comparable to resection
BCLC B (Bridging/downstaging)	TACE, TARE	TACE: 1-year OS > 90%, 5-year OS 20–35%. TARE: median OS ≈ 17 months.
BCLC B (Combination)	TACE + systemic agent	TACTICS (+sorafenib): median OS 36.2 mo. LAUNCH (+lenvatinib): 17.8 vs. 11.5 mo. EMERALD-1 (+durvalumab/bevacizumab): PFS * 15.0 vs. 8.2 mo.
BCLC C (Advanced HCC)	Systemic therapy	IMbrave150 (atezolizumab + bevacizumab): median OS 19.2 vs. 13.4 mo for sorafenib. HIMALAYA (durvalumab + tremelimumab): 5-year OS 19.6%.
Unresectable ICC *	TACE, TARE	Pooled median OS 14.2 months (TACE) and 13.5 months (TARE)

* Intrahepatic cholangiocarcinoma (ICC); microwave ablation (MWA); overall survival (OS); progression-free survival (PFS); radiofrequency ablation (RFA); stereotactic body radiotherapy (SBRT).

**Table 2:** Summary of Reported Clinical and Translational Advantages of PEDD.

Study	Outcome Measured	Reported Result
Microvalve infusion vs. end-hole DEM-TACE in solitary HCC	On-target microsphere deposition and tumor necrosis	88.7% vs. 55.3% on-target deposition; 89% vs. 56% tumor necrosis
Single-session SIS * DEM-TACE in unresectable HCC	Per-patient and per-lesion complete response rate	32% per-patient and 54% per-lesion CR after a single session
ARM * vs. conventional during Y-90 planning angiography	Need for prophylactic coil embolization and procedural metrics	Coil embolization eliminated; significantly reduced fluoroscopy time, procedure time, and radiation dose
ARM vs. conventional with tantalum microspheres in swine	Embolization efficiency	99.9% ± 1.0% vs. 72% ± 13% (*p* < 0.05)
Selective PEDD vs. end-hole conventional delivery in Oncopig liver tumor model	T/N * deposition ratio with embolic microspheres	T/N ratio improved from 2.7 to 4.2 (56% relative increase); trend toward reduced normal-tissue exposure (*p* = 0.090)
PEDD vs. conventional real-world cohort (matched analysis)	30-day inpatient visits after TACE and post-procedure fatigue	8.0% vs. 20.5% inpatient visits (61% reduction, *p* < 0.05); fatigue 20.9% vs. 26.4% (*p* < 0.05)
PEDD vs. conventional real-world cohort	Per-patient charge avoidance	~$7734 total avoidance per patient
ARC * vs. traditional coil-based nontarget protection during Y-90 TARE	Per-case savings from eliminating coil embolization	~$7000 in per-case savings
PEDD-mode anti-CEA CAR-T via TriNav in a CEA-positive liver metastasis patient	Intratumoral CAR-T delivery and clinical response	~5.2-fold increase in CAR-T delivery; 13-month complete response with no adverse events above grade 3
Pressure-enabled regional venous infusion of TLR9 agonist + anti-PD-1 vs. systemic delivery in murine pancreatic adenocarcinoma model	Intratumoral concentration of immunomodulatory agent	~3-fold higher intratumoral signal vs. systemic; reduced tumor burden in combination arm

* Surefire Infusion System (SIS); antireflux microcatheter (ARM); tumor-to-normal-tissue ratio; antireflux catheter (ARC).

**Table 3:** Current Clinical Trials of PEDD in Hepatopancreaticobiliary Oncology *.

Trial	Inclusion	Intervention
PERIO-03	Locally advanced pancreatic adenocarcinoma	Pancreatic retrograde venous infusion (PRVI) of SD-101 ± anti-PD-1
PEDIR	HCC, colorectal liver metastases	PEDD/TriNav vs. standard microcatheter during Y-90 radioembolization mapping
Fidelity	Primary and metastatic liver tumors	TriNav for ^99m^Tc-MAA mapping and Y-90 treatment
Lipiodol Deposition Using End-hole vs. Pressure-Modulated Delivery	HCC, neuroendocrine liver metastases	End-hole vs. TriNav for lipiodol TACE
PREDICTT	Primary and metastatic liver tumors	TriNav for Y-90 microsphere therapy; CT relative-enhancement analysis
Cryoablation and Arterial Infusion of SD-101 in Combination with Durvalumab and Tremelimumab	Advanced HCC	Cryoablation + hepatic arterial PEDD of SD-101 + durvalumab + tremelimumab

* Data compiled from ClinicalTrials.gov as of April 2026.

## Data Availability

No new data were created or analyzed in this study.

## Abbreviations

The following abbreviations are used in this manuscript:

BCLC	Barcelona Clinic Liver Cancer
TAE	Trans-arterial Embolization
CAR-T	Chimeric Antigen Receptor T-cell
TACE	Trans-arterial Chemoembolization
TARE	Trans-arterial Radioembolization
PEDD	Pressure-Enabled Drug Delivery
SIS	Surefire Infusion System
HCC	Hepatocellular Carcinoma
MDSCs	Myeloid-Derived Suppressor Cells
MVI	Microvalve Infusion
B-TACE	Balloon-occluded Trans-arterial Chemoembolization
ARC	Antireflux Catheter
IFP	Interstitial Fluid Pressure
C-TACE	Conventional Trans-arterial Chemoembolization
DEB-TACE	Drug-Eluting Bead Trans-arterial Chemoembolization
DSM-TACE	Degradable Starch Microspheres Trans-arterial Chemoembolization
Y-90	yttrium-90
MAA	Macro-Aggregated Albumin
^99m^Tc	Technetium-99 m

## References

[1] Garg T., Shrigiriwar A., Habibollahi P., Cristescu M., Liddell R.P., Chapiro J., Inglis P., Camacho J.C., Nezami N. (2022). Intra-arterial Therapies for the Management of Hepatocellular Carcinoma. Cancers.

[2] Sheth R.A., Hesketh R., Kong D.S., Wicky S., Oklu R. (2013). Barriers to drug delivery in interventional oncology. J. Vasc. Interv. Radiol..

[3] Cournoyer L., Liu Y., Jaroch D.B., Hullinger T.G., Cox B.F., Katz S.C., LaPorte J., Layman I.B., Ballarin A., Ghosh C.C. (2025). Pressure enabled drug delivery (PEDD) of nelitolimod increased therapeutic delivery, reduced immunosuppression, and improved efficacy in porcine and murine liver tumor models. Front. Oncol..

[4] Vaidya S., Tozer K.R., Chen J. (2008). An overview of embolic agents. Semin. Interv. Radiol..

[5] Scaffaro L.A., Kruel C.D.P., Stella S.F., Gravina G.L., Machado Filho G., Borges de Almeida C.P., Pinto L.C.P.F., Alvares-da-Silva M.R., Kruel C.R.P. (2015). Transarterial Embolization for Hepatocellular Carcinoma: A Comparison between Nonspherical PVA and Microspheres. BioMed Res. Int..

[6] Wáng Y.X.J., De Baere T., Idée J.M., Ballet S. (2015). Transcatheter embolization therapy in liver cancer: An update of clinical evidences. Chin. J. Cancer Res..

[7] Guan J.J., Golzarian J. (2023). Embolics review: Current options and agents in the pipeline. Vasc. Dis. Manag..

[8] Jipa A.M., Makary M.S. (2024). Locoregional Therapies for Hepatobiliary Tumors: Contemporary Strategies and Novel Applications. Cancers.

[9] Sacco R., Tapete G., Simonetti N., Sellitri R., Natali V., Melissari S., Cabibbo G., Biscaglia L., Bresci G., Giacomelli L. (2017). Transarterial chemoembolization for the treatment of hepatocellular carcinoma: A review. J. Hepatocell. Carcinoma.

[10] Ruff S.M., Chang J.Y., Xu M., Ejaz A.M., Dillhoff M., Pawlik T.M., Makary M.S., Rikabi A., Sukrithan V., Konda B. (2024). Trans-arterial embolization versus chemoembolization for neuroendocrine liver metastases: A propensity matched analysis. HPB.

[11] Crocetti L., Bozzi E., Scalise P., Bargellini I., Lorenzoni G., Ghinolfi D., Campani D., Balzano E., De Simone P., Cioni R. (2021). Locoregional Treatments for Bridging and Downstaging HCC to Liver Transplantation. Cancers.

[12] Fazlollahi F., Carfora A.D., King M., Wrasman E.S., Makary M.S. (2026). The State of the Art in Combination Locoregional and Systemic Treatment Strategies for Hepatocellular Carcinoma: Recent Advancements and Future Horizons. Curr. Oncol..

[13] Sakamoto I., Aso N., Nagaoki K., Matsuoka Y., Uetani M., Ashizawa K., Iwanaga S., Mori M., Morikawa M., Fukuda T. (1998). Complications associated with transcatheter arterial embolization for hepatic tumors. RadioGraphics.

[14] Makary M.S., Khandpur U., Cloyd J.M., Mumtaz K., Dowell J.D. (2020). Locoregional Therapy Approaches for Hepatocellular Carcinoma: Recent Advances and Management Strategies. Cancers.

[15] Singh R., Makary M.S. (2025). Locoregional Therapies for Hepatocellular Carcinoma with Portal Vein Tumor Thrombus. J. Gastrointest. Cancer.

[16] Mason M.C., Massarweh N.N., Salami A., Sultenfuss M.A., Anaya D.A. (2015). Post-embolization syndrome as an early predictor of overall survival after transarterial chemoembolization for hepatocellular carcinoma. HPB.

[17] Wu J., Song L., Zhao D.Y., Guo B., Liu J. (2014). Chemotherapy for transarterial chemoembolization in patients with unresectable hepatocellular carcinoma. World J. Gastroenterol..

[18] Lanza C., Ascenti V., Amato G.V., Pellegrino G., Triggiani S., Tintori J., Intrieri C., Angileri S.A., Biondetti P., Carriero S. (2025). All You Need to Know About TACE: A Comprehensive Review of Indications, Techniques, Efficacy, Limits, and Technical Advancement. J. Clin. Med..

[19] Makary M.S., Alexander J., Regalado L.E., Jalil S., Mumtaz K. (2024). Clinical outcomes of DEB-TACE in locally advanced hepatocellular carcinoma: A 5-year real world experience. PLoS ONE.

[20] Varela M., Real M.I., Burrel M., Forner A., Sala M., Brunet M., Ayuso C., Castells L., Montañá X., Llovet J.M. (2007). Chemoembolization of hepatocellular carcinoma with drug eluting beads: Efficacy and doxorubicin pharmacokinetics. J. Hepatol..

[21] Collettini F., Andrašina T., Reimer P., Schima W., Stroszczynski C., Lamprecht Y., Auer T.A., Rohan T., Wildgruber M., Gebauer B. (2025). Degradable starch microspheres transarterial chemoembolization (DSM-TACE) in patients with unresectable hepatocellular carcinoma: Results from the Prospective Multicenter Observational HepaStar Trial. Eur. Radiol..

[22] Saghafian Larijani R., Shabani Ravari N., Goodarzi N., Akhlaghpour S., Saghafian Larijani S., Rouini M.R., Dinarvand R. (2022). Current status of transarterial chemoembolization (TACE) agents in hepatocellular carcinoma treatment. J. Drug Deliv. Sci. Technol..

[23] Schicho A., Pereira P.L., Haimerl M., Niessen C., Michalik K., Beyer L.P., Stroszczynski C., Wiggermann P. (2017). Transarterial chemoembolization (TACE) with degradable starch microspheres (DSM) in hepatocellular carcinoma (HCC): Multi-center results on safety and efficacy. Oncotarget.

[24] Sergio A., Cristofori C., Cardin R., Pivetta G., Ragazzi R., Baldan A., Girardi L., Cillo U., Burra P., Giacomin A. (2008). Transcatheter arterial chemoembolization (TACE) in hepatocellular carcinoma (HCC): The role of angiogenesis and invasiveness. Am. J. Gastroenterol..

[25] Rozzanigo U., Gatti F., Luppi G., Costa L., Petralia B., Pravadelli C., Maioli I., Frisinghelli M., Fersino S., Berletti R. (2023). Chemoembolization with Degradable Starch Microspheres (DSM-TACE): Expanding indications in HCC multidisciplinary tumor board. Hepatoma Res..

[26] Wu F., Zeng H. (2025). A comparative study of DEB-TACE alone and DEB-TACE combined with targeted therapy and immunotherapy in the treatment of intermediate-advanced hepatocellular carcinoma. Cancer Treat. Res. Commun..

[27] Jiao H.Y., Yan X.M., Li J.X., Zhang Z.G. (2025). Combination therapy reduces transarterial chemoembolization resistance in advanced hepatocellular carcinoma. World J. Clin. Oncol..

[28] Lalenis C., Posa A., Lancellotta V., Lippi M., Marazzi F., Barbieri P., Cornacchione P., Fischer M.J., Tagliaferri L., Iezzi R. (2025). TACE Versus TARE in the Treatment of Liver-Metastatic Breast Cancer: A Systematic Review. Tomography.

[29] Sugumar K., Stitzel H., Wu V., Bajor D., Chakrabarti S., Conces M., Henke L., Lumish M., Mahipal A., Mohamed A. (2024). Outcomes of Hepatic Artery-Based Therapies and Systemic Multiagent Chemotherapy in Unresectable Colorectal Liver Metastases: A Systematic Review and Meta-analysis. Ann. Surg. Oncol..

[30] Sun H., Wang F., Yav S., Zhao D., Huo Z., Shuai J., Yan Z., Chen Y., Liu L., Wang W. (2025). Comparison of TACE alone versus TACE combined with synchronous ablation for neuroendocrine neoplasms with liver metastases. J. Cancer Res. Clin. Oncol..

[31] Remiszewski P., Topolewski P., Łaski D., Drobińska A. (2024). Outcomes of Bridging Therapy in Liver Transplantation for Hepatocellular Carcinoma. J. Clin. Med..

[32] Wang H., Liu E., Wu M., Gao H., Lv B., Chen G., Zeng X. (2026). Development of a Nomogram Model to Predict Post-Transplant Tumor Recurrence in Patients with Hepatocellular Carcinoma Beyond the Milan Criteria Undergoing TACE Bridging Therapy. J. Hepatocell. Carcinoma.

[33] Kwong A., Mehta N. (2021). Expanding the limits of liver transplantation for hepatocellular carcinoma: Is there a limit?. Clin. Liver Dis..

[34] Criss C.R., Makary M.S. (2024). Liver-Directed Locoregional Therapies for Neuroendocrine Liver Metastases: Recent Advances and Management. Curr. Oncol..

[35] Kim J.H., Kim J.H., Yoon H.K., Ko G.Y., Shin J.H., Gwon D.I., Ko H.K., Chu H.H., Kim S.H., Kim G.H. (2023). Transarterial chemoembolization for advanced hepatocellular carcinoma without macrovascular invasion or extrahepatic metastasis: Analysis of factors prognostic of clinical outcomes. Front. Oncol..

[36] Memon K., Lewandowski R.J., Kulik L., Riaz A., Mulcahy M.F., Salem R. (2011). Radioembolization for Primary and Metastatic Liver Cancer. Semin. Radiat. Oncol..

[37] Criss C.R., Makary M.S. (2023). Salvage locoregional therapies for recurrent hepatocellular carcinoma. World J. Gastroenterol..

[38] Knight G.M., Gordon A.C., Gates V., Talwar A., Riaz A., Salem R., Lewandowski R. (2023). Evolution of Personalized Dosimetry for Radioembolization of Hepatocellular Carcinoma. J. Vasc. Interv. Radiol..

[39] Garin E., Tselikas L., Guiu B., Chalaye J., Edeline J., de Baere T., Assenat E., Tacher V., Robert C., Terroir-Cassou-Mounat M. (2021). Personalised versus standard dosimetry approach of selective internal radiation therapy in patients with locally advanced hepatocellular carcinoma (DOSISPHERE-01): A randomised, multicentre, open-label phase 2 trial. Lancet Gastroenterol. Hepatol..

[40] Salem R., Johnson G.E., Kim E., Riaz A., Bishay V., Boucher E., Fowers K., Lewandowski R., Padia S.A. (2021). Yttrium-90 Radioembolization for the Treatment of Solitary, Unresectable HCC: The LEGACY Study. Hepatology.

[41] Mosconi C., Solaini L., Vara G., Brandi N., Cappelli A., Modestino F., Cucchetti A., Golfieri R. (2021). Transarterial Chemoembolization and Radioembolization for Unresectable Intrahepatic Cholangiocarcinoma—A Systemic Review and Meta-Analysis. Cardiovasc. Interv. Radiol..

[42] O’Donnell C.D.J., Majeed U., Rutenberg M.S., Croome K.P., Poruk K.E., Toskich B., Jin Z. (2025). Advancements in Locoregional Therapies for Unresectable Intrahepatic Cholangiocarcinoma. Curr. Oncol..

[43] Zane K.E., Makary M.S. (2021). Locoregional Therapies for Hepatocellular Carcinoma with Portal Vein Tumor Thrombosis. Cancers.

[44] Singal A.G., Llovet J.M., Yarchoan M., Mehta N., Heimbach J.K., Dawson L.A., Jou J.H., Kulik L.M., Agopian V.G., Marrero J.A. (2023). AASLD Practice Guidance on prevention, diagnosis, and treatment of hepatocellular carcinoma. Hepatology.

[45] Entezari P., Gabr A., Kennedy K., Salem R., Lewandowski R.J. (2021). Radiation Lobectomy: An Overview of Concept and Applications, Technical Considerations, Outcomes. Semin. Interv. Radiol..

[46] Mosconi C., Cappelli A., Pettinato C., Golfieri R. (2015). Radioembolization with Yttrium-90 microspheres in hepatocellular carcinoma: Role and perspectives. World J. Hepatol..

[47] Torkian P., Ragulojan R., Woodhead G., D’Souza D., Flanagan S., Golzarian J., Young S. (2022). Lung shunt fraction quantification methods in radioembolization: What you need to know. Br. J. Radiol..

[48] Feely M., Tondon R., Gubbiotti M., Stashek K.M., Numbere N., Huber A.R., Sharma A.K., Geller B.S., Salaria S.N., Gonzalez R.S. (2022). Gastrointestinal Tract Injury by Yttrium-90 Appears Largely Restricted to Resin Microspheres But Can Occur Years After Embolization. Am. J. Surg. Pathol..

[49] Ariffin A.B., Forde P.F., Jahangeer S., Soden D.M., Hinchion J. (2014). Releasing pressure in tumors: What do we know so far and where do we go from here? A review. Cancer Res..

[50] Stylianopoulos T., Jain R.K. (2013). Combining two strategies to improve perfusion and drug delivery in solid tumors. Proc. Natl. Acad. Sci. USA.

[51] Wu Z., Li C., Zhang S., Sun L., Hu J., Qiu B., Liu S., Hong Y., Chen T., Wang K. (2025). High interstitial fluid pressure enhances USP1-dependent KIF11 protein stability to promote hepatocellular carcinoma progression. J. Transl. Med..

[52] Kim A.Y., Miller A. (2016). Evaluation of Surefire’s precision direct-to-tumor embolization device to augment therapeutic response to intra-arterial, liver-directed therapies for patients with primary and secondary liver cancers. Expert Rev. Med. Devices.

[53] van den Hoven A.F., Lam M.G.E.H., Jernigan S., van den Bosch M.A.A.J., Buckner G.D. (2015). Innovation in catheter design for intra-arterial liver cancer treatments results in favorable particle-fluid dynamics. J. Exp. Clin. Cancer Res..

[54] Irie T., Kuramochi M., Takahashi N. (2013). Dense accumulation of lipiodol emulsion in hepatocellular carcinoma nodule during selective balloon-occluded transarterial chemoembolization: Measurement of balloon-occluded arterial stump pressure. Cardiovasc. Interv. Radiol..

[55] Inoue A., Ota S., Takaki K., Imai Y., Sato S., Watanabe S., Tomozawa Y., Iwai T., Murakami Y., Sonoda A. (2019). Change in hepatic hemodynamics assessed by hepatic arterial blood pressure and computed tomography during hepatic angiography with the double balloon technique. Jpn. J. Radiol..

[56] Arai H., Abe T., Takayama H., Toyoda M., Ueno T., Kakizaki S., Sato K. (2015). Safety and efficacy of balloon-occluded transcatheter arterial chemoembolization using miriplatin for hepatocellular carcinoma. Hepatol. Res..

[57] Ogawa M., Takayasu K., Hirayama M., Miura T., Shiozawa K., Abe M., Matsumoto N., Nakagawara H., Ohshiro S., Yamamoto T. (2016). Efficacy of a microballoon catheter in transarterial chemoembolization of hepatocellular carcinoma using miriplatin, a lipophilic anticancer drug: Short-term results. Hepatol. Res..

[58] Irie T., Kuramochi M., Kamoshida T., Takahashi N. (2016). Selective balloon-occluded transarterial chemoembolization for patients with one or two hepatocellular carcinoma nodules: Retrospective comparison with conventional super-selective TACE. Hepatol. Res..

[59] Lucatelli P., De Rubeis G., Trobiani C., Ungania S., Rocco B., Zilahi De Gyurgyokai S., Masi M., Pecorella I., Cappelli F., Lai Q. (2022). In vivo comparison of micro-balloon interventions (MBI) advantage: A retrospective cohort study of DEB-TACE versus b-TACE and of SIRT versus b-SIRT. Cardiovasc. Interv. Radiol..

[60] Golfieri R., Bezzi M., Verset G., Fucilli F., Mosconi C., Cappelli A., Paccapelo A., Lucatelli P., Magand N., Rode A. (2021). Retrospective European multicentric evaluation of selective transarterial chemoembolisation with and without balloon-occlusion in patients with hepatocellular carcinoma: A propensity score matched analysis. Cardiovasc. Interv. Radiol..

[61] Lucatelli P., De Rubeis G., Rocco B., Basilico F., Cannavale A., Abbatecola A., Nardis P.G., Corona M., Brozzetti S., Catalano C. (2021). Balloon occluded TACE (B-TACE) vs. DEM-TACE for HCC: A single center retrospective case control study. BMC Gastroenterol..

[62] Irie T., Takahashi N., Kamoshida T., Kashimura J., Ariga H. (2020). Balloon-Occluded Trans-Arterial Chemo-Embolization Technique with Repeated Alternate Infusion of Cisplatin Solution and Sparse Gelatin Slurry (RAIB-TACE) for Large Hepatocellular Carcinoma Nodules More than 7 cm in Diameter. Biomed. Res. Int..

[63] Embolx (2018). Sniper Balloon Occlusion Microcatheter: Product Information.

[64] Gwon D.I., Kim J.H., Chu H.H., Kim G.H., Kim J., Im B.S., Ko G.-Y., Yoon H.-K. (2026). Lipiodol-based balloon-occluded transcatheter arterial chemoembolization can be a curative treatment option for hepatocellular carcinoma. J. Vasc. Interv. Radiol..

[65] Rose S.C., Kikolski S.G., Chomas J.E. (2013). Downstream hepatic arterial blood pressure changes caused by deployment of the Surefire antireflux expandable tip. Cardiovasc. Interv. Radiol..

[66] Rose S.C., Narsinh K.H., Newton I.G. (2017). Quantification of blood pressure changes in the vascular compartment when using an anti-reflux catheter during chemoembolization versus radioembolization: A retrospective case series. J. Vasc. Interv. Radiol..

[67] Jaroch D.B., Liu Y., Kim A.Y., Katz S.C., Cox B.F., Hullinger T.G. (2024). Intra-arterial pressure-enabled drug delivery significantly increases penetration of glass microspheres in a porcine liver tumor model. J. Vasc. Interv. Radiol..

[68] Jain R.K. (1994). Barriers to drug delivery in solid tumors. Sci. Am..

[69] Shankara Narayanan J.S., Vicente D.A., Ray P., Chai L.F., Erdem S., Carr M.J., Capacio B.A., Cox B.F., Jaroch D.B., Katz S.C. (2020). Pressure-enabled delivery of gemcitabine in an orthotopic pancreatic cancer mouse model. Surgery.

[70] Jaroch D.B., Liu Y., Kim A.Y., Katz S.C., Cox B.F., Hullinger T.G. (2025). Pressure-enabled drug delivery significantly increases intra-arterial delivery of embolic microspheres to liver tumors in a porcine model. J. Vasc. Interv. Radiol..

[71] Titano J.J., Fischman A.M., Cherian A., Tully M., Stein L.L., Jacobs L., Rubin R.A., Bosley M., Citron S., Joelson D.W. (2019). End-hole versus microvalve infusion catheters in patients undergoing drug-eluting microspheres-TACE for solitary hepatocellular carcinoma tumors: A retrospective analysis. Cardiovasc. Interv. Radiol..

[72] Pasciak A.S., McElmurray J.H., Bourgeois A.C., Heidel R.E., Bradley Y.C. (2015). The impact of an antireflux catheter on target volume particulate distribution in liver-directed embolotherapy: A pilot study. J. Vasc. Interv. Radiol..

[73] Kim A.Y., Frantz S., Krishnan P., DeMulder D., Caridi T., Lynskey G.E., Spies J.B. (2017). Short-term imaging response after drug-eluting embolic trans-arterial chemoembolization delivered with the Surefire Infusion System for the treatment of hepatocellular carcinoma. PLoS ONE.

[74] Fischman A.M., Ward T.J., Patel R.S., Arepally A., Kim E., Nowakowski F.S., Lookstein R.A. (2014). Prospective, randomized study of coil embolization versus Surefire Infusion System during yttrium-90 radioembolization with resin microspheres. J. Vasc. Interv. Radiol..

[75] van den Hoven A.F., Prince J.F., Samim M., Arepally A., Zonnenberg B.A., Lam M.G.E.H., van den Bosch M.A.A.J. (2014). Posttreatment PET-CT-confirmed intrahepatic radioembolization performed without coil embolization, by using the antireflux Surefire Infusion System. Cardiovasc. Interv. Radiol..

[76] Arepally A., Chomas J., Kraitchman D., Hong K. (2013). Quantification and reduction of reflux during embolotherapy using an antireflux catheter and tantalum microspheres: Ex vivo analysis. J. Vasc. Interv. Radiol..

[77] Pham R., Swisher A.R., Theodory B., Kessler J. (2023). Large hepatic biloma after bland hepatic arterial embolization using antireflux catheter. Radiol. Case Rep..

[78] Rose S.C., Narsinh K.H., Isaacson A.J., Fischman A.M., Golzarian J. (2019). The Beauty and Bane of Pressure-Directed Embolotherapy: Hemodynamic Principles and Preliminary Clinical Evidence. Am. J. Roentgenol..

[79] Gupta D., Liu Y., Miller-Rosales C., Wei F., Tuttle E., Byers E., Marshak R., Marshall R. (2026). Clinical outcomes of pressure-enabled drug delivery for trans-arterial chemoembolization and radioembolization. J. Comp. Eff. Res..

[80] Cook K., Gupta D., Liu Y., Miller-Rosales C., Wei F., Tuttle E., Katz S.C., Marshak R., Kim A.Y. (2024). Real-world evidence of Pressure-Enabled Drug Delivery for trans-arterial chemoembolization and radioembolization among patients with hepatocellular carcinoma and liver metastases. Curr. Med. Res. Opin..

[81] Morshedi M.M., Bauman M., Rose S.C., Kikolski S.G. (2015). Yttrium-90 resin microsphere radioembolization using an antireflux catheter: An alternative to traditional coil embolization for nontarget protection. Cardiovasc. Interv. Radiol..

[82] Thorn M., Guha P., Cunetta M., Espat N.J., Miller G., Junghans R.P., Katz S.C. (2016). Tumor-associated GM-CSF overexpression induces immunoinhibitory molecules via STAT3 in myeloid-suppressor cells infiltrating liver metastases. Cancer Gene Ther..

[83] Burga R.A., Thorn M., Point G.R., Guha P., Nguyen C.T., Licata L.A., DeMatteo R.P., Ayala A., Joseph Espat N., Junghans R.P. (2015). Liver myeloid-derived suppressor cells expand in response to liver metastases in mice and inhibit the anti-tumor efficacy of anti-CEA CAR-T. Cancer Immunol. Immunother..

[84] Ghosh C.C., Heatherton K.R., O’Connell K.P., Alexander I.S., Greer D.A., LaPorte J., Guha P., Cox B.F., Katz S.C. (2022). Regional infusion of a class C TLR9 agonist enhances liver tumor microenvironment reprogramming and MDSC reduction to improve responsiveness to systemic checkpoint inhibition. Cancer Gene Ther..

[85] DaSilva N.A., Barlock B.J., Guha P., Ghosh C.C., Trebino C.E., Camberg J.L., Katz S.C., Rowley D.C. (2021). Proteomic signatures of myeloid derived suppressor cells from liver and lung metastases reveal functional divergence and potential therapeutic targets. Cell Death Discov..

[86] Katz S.C., Burga R.A., McCormack E., Wang L.J., Mooring W., Point G.R., Khare P.D., Thorn M., Ma Q., Stainken B.F. (2015). Phase I Hepatic Immunotherapy for Metastases study of intra-arterial chimeric antigen receptor-modified T-cell therapy for CEA+ liver metastases. Clin. Cancer Res..

[87] Katz S.C., Hardaway J., Prince E., Guha P., Cunetta M., Moody A., Wang L.J., Armenio V., Espat N.J., Junghans R.P. (2020). HITM-SIR: Phase Ib trial of intra-arterial chimeric antigen receptor T-cell therapy and selective internal radiation therapy for CEA+ liver metastases. Cancer Gene Ther..

[88] Katz S.C., Moody A.E., Guha P., Hardaway J.C., Prince E., LaPorte J., Stancu M., Slansky J.E., Jordan K.R., Schulick R.D. (2020). HITM-SURE: Hepatic immunotherapy for metastases phase Ib anti-CEA CAR-T study utilizing pressure enabled drug delivery. J. Immunother. Cancer.

[89] Capacio B.A., Shankara Narayanan J.S., Vicente D.A., Liu Y., LaPorte J.P., Cox B.F., Jaroch D.B., Katz S.C., White R.R. (2023). Pressure-Enabled Drug Delivery (PEDD) of a class C TLR9 agonist in combination with checkpoint inhibitor therapy in a murine pancreatic cancer model. Surgery.

[90] Ghosh C.C., Cournoyer L., Liu Y., Ballarin A., Layman I.B., LaPorte J., Morrissey M., Fraser K., Perati S., Cox B.F. (2024). Subcutaneous checkpoint inhibition is equivalent to systemic delivery when combined with nelitolimod delivered via pressure-enabled drug delivery for depletion of intrahepatic myeloid-derived suppressor cells and control of liver metastases. J. Immunother. Cancer.

[91] Sullivan K.M., Jiang X., Guha P., Lausted C., Carter J.A., Hsu C., Labadie K.P., Kohli K., Kenerson H.L., Daniel S.K. (2022). Blockade of interleukin 10 potentiates antitumour immune function in human colorectal cancer liver metastases. Gut.

[92] van Roekel C., van den Hoven A.F., Bastiaannet R., Bruijnen R.C.G., Braat A.J.A.T., de Keizer B., Lam M.G.E.H., Smits M.L.J. (2021). Use of an anti-reflux catheter to improve tumor targeting for holmium-166 radioembolization: A prospective, within-patient randomized study. Eur. J. Nucl. Med. Mol. Imaging.

[93] Berman C., Krishnasamy V.P. (2026). Pressure modification during hepatic tumor embolization: Principles, mechanism of action, and current evidence. CVIR Oncol..

[94] Chai L.F., Hardaway J.C., Heatherton K.R., O’Connell K.P., Lopes M.C., Rabinowitz B.A., Ghosh C.C., Guha P., Jaroch D., Cox B.F. (2021). Regional delivery of anti-PD-1 agent for colorectal liver metastases improves therapeutic index and anti-tumor activity. Vaccines.

